# Wiskott-Aldrich Syndrome Presenting with JMML-Like Blood Picture and Normal Sized Platelets

**DOI:** 10.1155/2016/8230786

**Published:** 2016-05-31

**Authors:** Rajesh B. Patil, Chandrakala Shanmukhaiah, Farah Jijina, Shailesh Bamborde, Nilesh Wasekar, Manoj Toshniwal, Aniket Mohite, Vinod Patil

**Affiliations:** Dr. J. C. Patel Department Of Clinical Hematology, Seth GS Medical College & KEM Hospital, Mumbai, Maharashtra 400012, India

## Abstract

*Objective*. The aim of this paper is to report the case of Wiskott-Aldrich syndrome (WAS) that presented with unusual laboratory features.* Clinical Presentation and Intervention*. Male neonate admitted with symptoms related to thrombocytopenia, whose initial diagnosis was considered as neonatal alloimmune thrombocytopenia and JMML (juvenile myelomonocytic leukemia) but subsequently diagnosis was confirmed as WAS.* Conclusion*. This case shows that a suspicion of WAS is warranted in the setting of neonatal thrombocytopenia with JMML-like blood picture and normal sized platelets.

## 1. Introduction

Although thrombocytopenia is relatively rare in the general newborn population, it occurs more frequently in patients admitted to neonatal intensive care units. Platelet count < 50 × 10^9^/L is considered as severe thrombocytopenia. Severe neonatal thrombocytopenia is uncommon in the general healthy newborn population, with a reported incidence between 0.14% and 0.24% [1, 2]. Severe neonatal thrombocytopenia (platelet count < 50 × 10^9^/L) is associated with bleeding and, potentially, significant morbidity, although there is poor correlation between platelet count and incidence of bleeding. As a result, it is important to identify at-risk infants and if needed initiate therapy to prevent associated complications.

Causes of thrombocytopenia can be classified by several different methods including platelet size (i.e., large, normal, and small), mode of acquisition (congenital or acquired), early (<72 hours of age) or late (≥72 hours of age) onset, gestational age, or underlying pathologic mechanisms of decreased production or increased destruction. The case series reports that a cause of thrombocytopenia is identified in about 50% to 75% of neonates [3, 4].

## 2. Case Report

A two-day-old male neonate, first birth by order born out of a nonconsanguineous marriage, was admitted in NICU with petechial rashes all over the body. Baby was born full term and healthy and there was no history of birth trauma present. There were no history of fever, respiratory distress, or signs of sepsis and no maternal history of preeclampsia, thrombocytopenia, or bleeding tendency. Patient's examination was unremarkable except for petechial lesions. Complete hemogram showed Hb of 15.3 g/dL, WBC of 19 × 10^9^/L, PLT of 18 × 10^9^/L, and MPV of 8.5 fL with shift to the left and presence of NRBCs (Table 1).

Mother's hemogram, transfusion transmitted disease workup, and tests detecting antibodies against platelet antigens (antiplatelet antibodies) were not contributory. Baseline immunoglobulin levels were normal (IgG Levels 317 mg/dL, IgA 14 mg/dL, and IgM 87 mg/dL). A diagnosis of neonatal alloimmune thrombocytopenia was considered and IVIG 1 gm/kg was given along with platelets transfusion. Human platelet antigen genotyping of parents was done but no incompatibility was found. Baby's platelet increased up to 183 × 10^9^/L on day 3 of IVIG and he was discharged.

**Figure 1:**
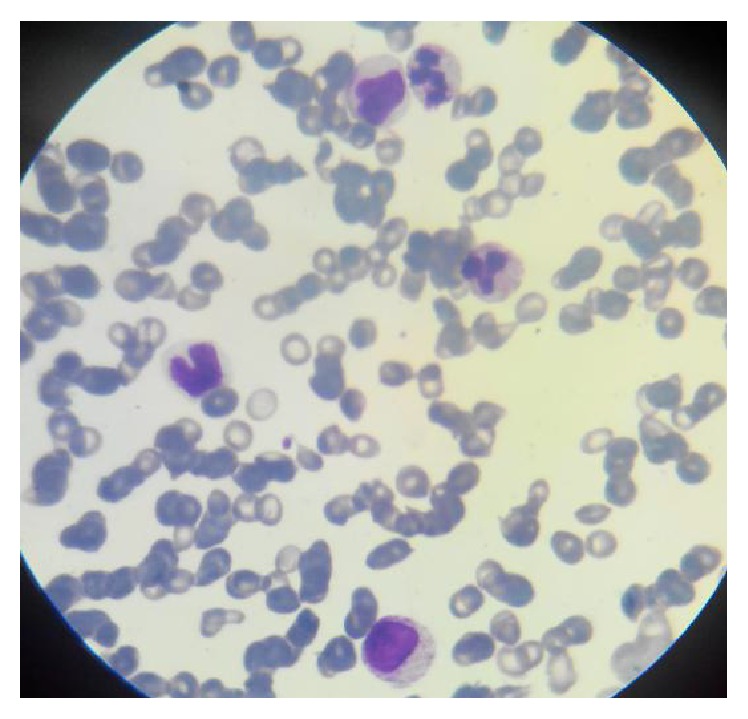
Peripheral smear showing monocytes, myeloid precursor, and normal sized platelet.

On day 25 of life, baby was again admitted with similar symptoms along with bloody stools. Patient's examination revealed pallor, petechial lesions and spleen was palpable 2 cm below left costal margin, and the rest of history and examination was unremarkable. Complete hemogram showed Hb of 11.2 g/dL, WBC of 22.8 × 10^9^/L, PLT of 20 × 10^9^/L, MPV of 8.3 fL, and NRBCS of 8/100 WBCs, and platelet morphology was normal (Table 1) (Figure 1).

Workup for infections and sepsis was negative. Baby's FISH for BCR-ABL was negative. In view of pallor, splenomegaly, persistent leukocytosis, monocytosis, and myeloid and erythroid precursors in the peripheral blood, a diagnosis of JMML was considered (see the following).

 Minimal diagnostic criteria for JMML (adopted by the International JMML Working Group and by EWOG-MDS) are described as follows.

 (i) Suggestive clinical features are as follows:

 Hepatosplenomegaly.
 Lymphadenopathy.
 Pallor.
 Fever.
 Skin rash.

(ii) Laboratory criteria (all three must be met) are as follows:

1. Persistent peripheral blood monocytosis (>1 × 10^9^/L).
2. No Philadelphia chromosome or* BCR-ABL* fusion gene.
3. <20% myeloblasts or monoblasts in the marrow.

(iii) Further criteria to be met (need to fulfill at least two) are as follows:

1. Increased hemoglobin F (corrected for age).
2. Immature myeloid precursors on the peripheral blood smear.
3. Peripheral blood white blood cell count >1 × 10^10^/L.
4. Clonal cytogenetic abnormalities (including monosomy 7).
5. GM-CSF hypersensitivity of myeloid progenitors (*in vitro* test).

Bone marrow examination showed normocellular marrow with eosinophilia and adequate megakaryocytes. Cytogenetics and FISH for monosomy 7/Del 7 revealed no abnormality. HbF level was in normal range (HbF 34.7%). Patient's lymphocyte subset analysis and repeat immunoglobulin levels were also normal and no mutation was detected on RAS pathway PCR analysis. Due to inconclusive reports and unavailability of other specific JMML diagnostic workup such as spontaneous colony assay, GM-CSF hypersensitivity and diagnostic dilemma persisted. Meanwhile, patients had received a repeat course of IVIG and platelets transfusion, and platelet count increased to 150 × 10^9^/L on day 3 of IVIG.

**Figure 2:**
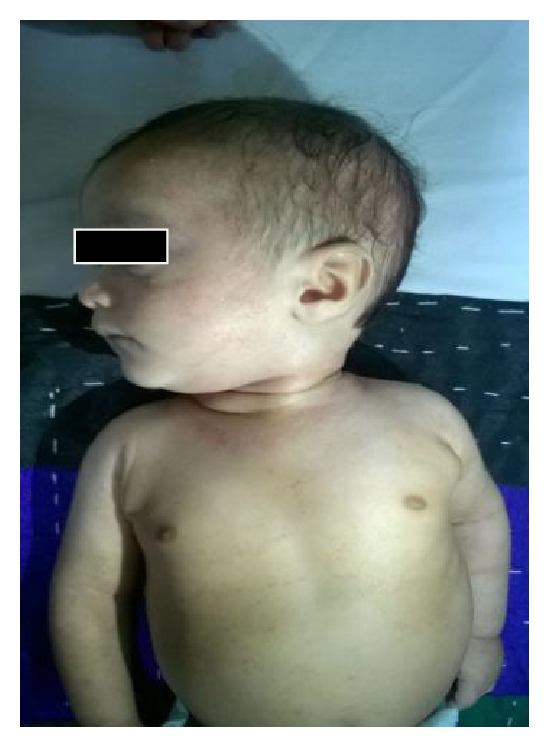
Showing maculopapular rash over baby's face and trunk.

For the next 2 months of follow-up, patient was intermittently symptomatic for petechial lesions with platelet count within range of 20–30 × 10^9^/L. High WBC count with shift to the left and eosinophilia were persistent. At 4 months of life, on routine OPD visit maculopapular rash was noted on baby's forehead and cheek (Figure 2).

These spots prompted us to reconsider diagnosis of Wiskott-Aldrich syndrome in the baby. Hence baby's complete gene sequencing for WAS gene was done, and it showed known pathogenic mutation c.37C>T (p.R13X) in WAS gene confirming diagnosis of Wiskott-Aldrich syndrome.

## 3. Discussion

Wiskott-Aldrich syndrome is a primary immunodeficiency disorder which presents with classic triad of thrombocytopenia with small platelets, recurrent infections, and eczema. Our case was atypical and unusual due to (1) blood picture like JMML and normal platelet morphology on peripheral smear, (2) normal MPV on repeated testing, and (3) good response to platelets transfusion and IVIG in on multiple occasions.

Review of literature reveals occasional rare cases of WAS, which may present with blood picture of JMML and normal MPV [5]. Thrombocytopenia in WAS does not respond to IVIG; however there are reports in literature supporting that IVIG administration can improve response to platelet transfusion and may normalize the platelet count temporarily [6].

## 4. Conclusion

Our case concludes that a suspicion of WAS is warranted in the setting of neonatal thrombocytopenia with JMML-like blood picture and normal sized platelets.

## Figures and Tables

**Table 1:** Showing patient's complete hemograms in two admissions.

Sr number	Parameters	On day 2 of life	On day 25 of life
1	*Hb *	15.3 g/dL	11.2 g/dL
2	MCV	88 fL	79.7 fL
3	*Total WBC count *	19.8 × 10^9^/L	22.8 × 10^9^/L
4	Polymorphs	46%	40%
5	Lymphocytes	15%	18%
6	Monocytes	15%	11%
7	Myelocytes + Metamyelocytes	8%	6%
8	Band forms	2%	2%
9	Atypical blast-like cells	—	2%
10	Eosinophils	14%	21%
11	*Platelet count *	18 × 10^9^/L	20 × 10^9^/L
12	MPV	8.5 fL	8.3 fL
13	*Absolute Monocyte Count (AMC)*	*2.97 × 10* ^*9*^ */L*	*2.5 × 10* ^*9*^ */L*
14	*HbF*	*Not done*	*34.7%*
